# Heterogeneity and metabolic diversity among *Enterococcus* species during long-term colonization

**DOI:** 10.1128/spectrum.03160-24

**Published:** 2025-06-12

**Authors:** Philip A. Karlsson, Taoran Zhang, Josef D. Järhult, Enrique Joffré, Helen Wang

**Affiliations:** 1 Department of Medical Biochemistry and Microbiology, Uppsala University 8097, Uppsala, Sweden; 2 Department of Medical Sciences, Zoonosis Science Center, Uppsala University 8097, Uppsala, Sweden; 3 Department of Microbiology, Tumor and Cell Biology, Karolinska Institute, Stockholm, Sweden; Quest Diagnostics Nichols Institute, Chantilly, Virginia, USA

**Keywords:** bacteriology, molecular biology, antibiotic resistance, *Enterococcus*, plasmids, clinical microbiology, PhP, UTI, ICU, polyclonal

## Abstract

Urinary tract infections (UTIs), traditionally dominated by Gram-negative pathogens, are increasingly complicated by antimicrobial-resistant *Enterococcus* spp. in hospital settings, particularly during the use of indwelling catheters. This study screened urine samples from 210 catheterized intensive care unit patients at Uppsala University Hospital (June 2020–September 2021), identifying 39 unique PhenePlate™-RF types across *E. faecium*, *E. faecalis*, and *E. durans*. *E. faecium* isolates showed considerable diversity, primarily within clonal complex 17 (CC17), known for its virulence and antibiotic resistance. We identified multiple lineages and sequence types (STs), such as in patient HWP143, who had isolates from both ST80 and ST22 (an ancestral CC17 lineage). Notably, metabolic adaptations, such as increased L-arabinose metabolism, and shifts in antibiotic resistance were observed. Variations and similarities in plasmid content between individual lineages suggest horizontal gene transfer. *E. faecalis* isolates exhibited less diversity, but still significant metabolic variability across patients and mixed infections, as seen in patient HWP051, colonized by both ST16 (CC58) and ST287. *E. durans*, though less common, shared important metabolic traits with *E. faecium* and displayed polyclonal characteristics, highlighting its potential role in UTIs and the complexity of enterococcal infections. *E. durans* was sometimes misidentified, underlining the need for accurate identification methods. This research underscores the importance of understanding genetic and metabolic diversity, plasmid variations, and horizontal gene transfer (HGT) in *Enterococcus* spp., which influence antibiotic resistance, virulence, and ultimately, treatment outcomes.

**IMPORTANCE:**

Our study, performed in Uppsala University Hospital, Sweden, uncovers novel insights into the genetic and metabolic diversity of *Enterococcus* species, focusing on *E. faecium*, *E. faecalis*, and *E. durans*. Unlike prior studies, which often have focused on single lineages, we reveal multiple clones and lineages within individual catheterized intensive care unit patients, including clones from clonal complex 17 and the emerging sequence type (ST) 192, highlighting notable metabolic adaptations and shifts in antibiotic resistance. The detection of mixed colonization with varied ST types and *E. durans* misidentification by matrix-assisted laser desorption/ionization time-of-flight mass spectrometry further emphasizes the challenges in *Enterococcus* species identification. Our findings have significant implications for understanding the complexity of *Enterococcus* infections, stressing the need to consider genetic and metabolic diversity to improve disease management and treatment outcomes.

## INTRODUCTION

Urinary tract infections (UTIs) are among the most prevalent bacterial diseases, posing a considerable burden to public health, particularly in the face of rising antimicrobial resistance (1). Risk factors for UTIs include pregnancy, anatomical, and functional abnormalities of the urinary tract and the use of indwelling urinary catheters (2). Catheters are the major risk factor for healthcare-acquired infections, and catheter-associated UTIs (CAUTIs) remain a prominent health challenge for patients in intensive care (3).

Gram-negative pathogens have traditionally been considered the primary causative agents of UTIs (4); however, recent studies reveal an increasing prevalence of *Enterococcus* species in urinary infections (5, 6). Historically, uropathogenic *Escherichia coli* (UPEC) have been responsible for 75%–85% of all UTIs. Although Gram-positive bacteria constitute less than 15% of community-acquired UTIs, they are more frequently associated with nosocomial infections (7, 8). *Enterococcus*, naturally resistant to many first-line antibiotics and capable of forming biofilms, contributes to immune evasion and treatment failure (9). Certain *Enterococcus* strains can acquire high-level resistance to ß-lactams, aminoglycosides, glycopeptides, and even combined antibiotic therapies. In Scandinavia, vancomycin (VAN)-resistant *Enterococcus* are rarely found but are causing outbreaks at an increasing and alarming speed (10–12). Ampicillin (AMP)-resistant *E. faecium* is gradually more common in healthcare-associated infections (13). In the United States, *Enterococcus* spp. were responsible for 12% of all CAUTIs between 2006 and 2007 (14). This is likely due to their ability to elicit proinflammatory responses in the bladder and form biofilm (15), which enables them to persist and cause chronic infections.

Bacterial populations are inherently heterogeneous, providing selective advantages during environmental changes and profoundly influencing clinical outcomes (16). Recent studies have demonstrated that *E. faecalis* exhibits heterogeneity in adhesion and biofilm formation. These key virulence properties may contribute to prolonged hospital stays and treatment failure (17), but it remains unknown to what extent heterogeneity or polymicrobial colonization affects patient outcomes (18).

In this study, we focus on bacterial urine colonizers among catheterized critically ill patients with severe acute respiratory syndrome coronavirus 2 infection. Early studies from similar cohorts have revealed that severely ill patients have a significantly higher risk of acquiring bacterial co-infections, especially with resistant strains, due to factors such as increased antibiotic use, utilization of urinary catheters, and the administration of immunosuppressive drugs (19, 20). A 2022 study in Madrid investigated 87 COVID-19 patients, with 89.6% having acquired UTIs and 67.9% being related to CAUTIs. *Enterococcus* was identified as the dominant genus, representing 47.4% of the isolates in the study cohort (5). However, the role of heterogeneity in these conditions remains unknown.

Distinguishing between species and clinically relevant strains of *Enterococcus* can be challenging, and their rapid emergence and importance demand a fast and accurate screening method (21, 22). In this study, we utilized the PhenePlate-RF (PhP-RF) system, a method for strain screening that provides a biochemical fingerprint for multiple clinical *Enterococcus* species, first introduced by Kühn et al. (23). This method is fast, highly reproducible, and possesses strong discriminatory power. The PhP-RF system has been successfully used and validated for screening *Enterococcus* in multiple environments, including the food chain and sewage water (24). However, its application to clinical *Enterococcus* isolates has been rare (22). Our study employs the PhP-RF system to investigate the heterogeneity and dynamics of clinical *Enterococcus* during urine colonization.

## RESULTS

From June 2020 to September 2021, urine samples were collected from 210 catheterized patients undergoing intensive care at Uppsala University Hospital. These patients were treated across multiple intensive care unit (ICU) wards, and their characteristics are described in our previous work (6, 25). A timeline outlining the patients’ length of stay and the collection points can be found in the supplementary material (Fig. S1). Sixteen enterococcal isolates per urine sample (29 separate urine samples) were screened using the PhP-RF system. In total, 456 colonies (*E. faecium*: *n* = 245, *E. faecalis*: *n* = 194, and *E. durans*: *n* = 17) were assessed, and 39 PhP-types (*E. faecium*: *n* = 25, *E. faecalis*: *n* = 8, and *E. durans*: *n* = 6) were identified using the pair group method with arithmetic mean (PGMA) clustering approach. One representative isolate per PhP-type and patient isolation day was confirmed with matrix-assisted laser desorption/ionization time-of-flight (MALDI-TOF) mass spectrometry and whole-genome sequencing (WGS) (Fig. S2). The PhP-RF system successfully separated between *E. faecalis* and *E. faecium*, but grouped *E. durans* isolates among *E. faecium* due to similar metabolic profiles (Fig. S6).

### *Enterococcus faecium* isolates showed high heterogeneity

**Fig 1:**
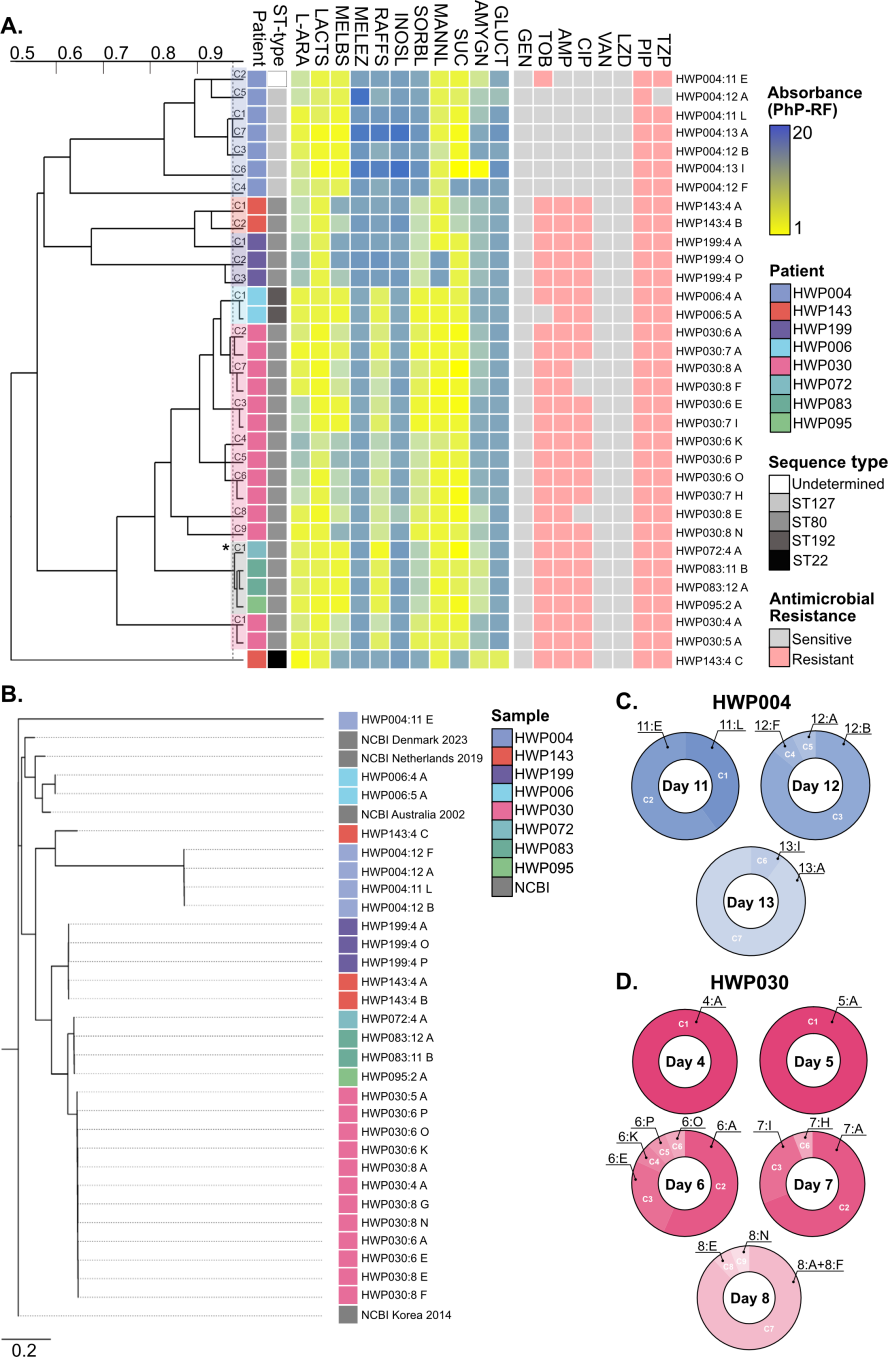
Heterogeneity of *E. faecium* in the patient cohort. (A) Dendrogram derived from unweighted pair group method with arithmetic mean (UPGMA) clustering of PhP-RF data (absorbance values at 620 nm). The blue-yellow gradient represents a scale from low (blue) to high (yellow) metabolism. Isolates with the most recent branching point above the 0.975 cut-off value (dotted line) are grouped into a PhP-type. If more than one isolate per patient was isolated, these clusters were numbered within that species (indicated by color). If isolates from different patients were grouped into the same PhP-cluster, this was indicated by an asterisk (*). For a full list of metabolic reagents, refer to the material and methods or supplementary data. Phenotypic resistance (pink) and susceptibility (gray) were assessed using broth microdilutions according to EUCAST. ST types were determined based on Illumina WGS. (B) Phylogenetic tree based on single nucleotide polymorphism (SNP) comparison of Illumina WGS data. Four NCBI strains are included as comparative references based on diversified historical and geographic backgrounds. The tree was made using the NCBI Korean isolate (GCF_002007625) as the main reference. (C) PhP-type cluster diversity (proportion) is based on the number of colonies from the total number screened (*n* = 16) for isolates from patient HWP004. (D) PhP-type cluster diversity (proportion) is based on the number of colonies from the total number screened (*n* = 16) for isolates from patient HWP030.

*E. faecium* isolates were grouped into two major PhP-branches, seemingly related to raffinose metabolism. The first branch included all strains from patients HWP004 and HWP199, and two isolates (HWP143:4A/B) from patient HWP143 (Fig. 1A). HWP143:4C (representing 81% of the HWP143:4 population) did not group with either of these branches, standing alone in the tree, likely due to its inability to degrade sucrose. This separation among HWP143 isolates was also observed genetically, collectively indicating heterogeneity among these isolates (Fig. 1B). This genetic separation is further supported by the isolates carrying different plasmids (Fig. S3). HWP004:11E could not have its ST type determined through the chosen method but distinctly grouped among ST127 isolates, potentially suggesting a new ST variant or mixed sample. All HWP004 isolates belonged to different PhP-types, yet HWP004:11E was the only HWP004-isolate grouping differently phylogenetically (Fig. 1). The different PhP types across the different days within one patient were presented as proportions of subpopulations (Fig. 1C and D). HWP004:11E was the only HWP004 strain demonstrating resistance to tobramycin (TOB) (Fig. 1). While implausibly related to TOB resistance, HWP004:11E did not carry the repUS15 plasmid, which was present in all other HWP004 strains (Fig. S3). Instead, HWP004:11E carried the rep1 plasmid, which was not found in HWP004:12A and HWP004:12F but was present in HWP004:13A and HWP004:13I. Unlike all other HWP004 strains, HWP004:12A did not tolerate piperacillin-tazobactam (TZP). Multi-locus sequence typing (MLST) could not confidently assign a sequence type for HWP004:11E due to incomplete allele matches, and results for this isolate should hence be cautiously interpreted.

Another interesting observation can be seen in patient HWP030, where a metabolic change occurred from HWP030:5A to all the strains from collection day 6 (Fig. 1A). This difference exists despite the strains having genetically near-identical core genomes (Fig. 1B) (average nucleotide identity for orthologous regions > 99.97%). The patient underwent further changes by collection day 8, where 93% of the population (clusters C2 and C3, Fig. 1D) lost its resistance to ciprofloxacin (CIP) without altering its PhP-type. Interestingly, the replicon identity of rep11a and rep14a fluctuated between 95% and 100% across the isolates in HWP030 (Fig. S3). Similar plasmid identity variations (but for rep14a) could be observed in HWP083:11A/B and HWP199:4A/O/P.

Overall, *E. faecium* isolates showed poor metabolic activity for melezitose, inositol, and gluconate, but good metabolic activity for L-arabinose, lactose, melibiose, mannitol, and sucrose. More varied results were observed for raffinose, sorbitol, and amygdalin. For instance, none of the isolates from HWP004, nor HWP143:4C, could metabolize sorbitol. The only obvious commonality between the HWP004 isolates and HWP143:4C, except for them being phylogenetically closer to each other than to other strains, was that these were the only isolates not carrying plasmid replicon 11a. While raffinose metabolism was fairly stable among isolates within a single patient, amygdalin metabolism varied greatly even among isolates within the same patient. Notably, in patient HWP004, the smallest subpopulation during the last collection day, represented by HWP004:13I (cluster 6, 6.25%, Fig. 1C), had acquired a remarkably strong metabolism of amygdalin. Two additional significant examples are found in patient HWP199, where HWP199:4A uniquely metabolizes mannitol, and in patient HWP143, where HWP143:4C alone metabolizes mannitol, amygdalin, and gluconate in the absence of melibiose metabolism. It is noteworthy that all three strains of HWP143 were isolated on the same day, and that HWP143:4C possessed substantially fewer antimicrobial resistance (AMR) genes (Fig. S5).

### Low heterogeneity among *Enterococcus faecalis* isolates

**Fig 2:**
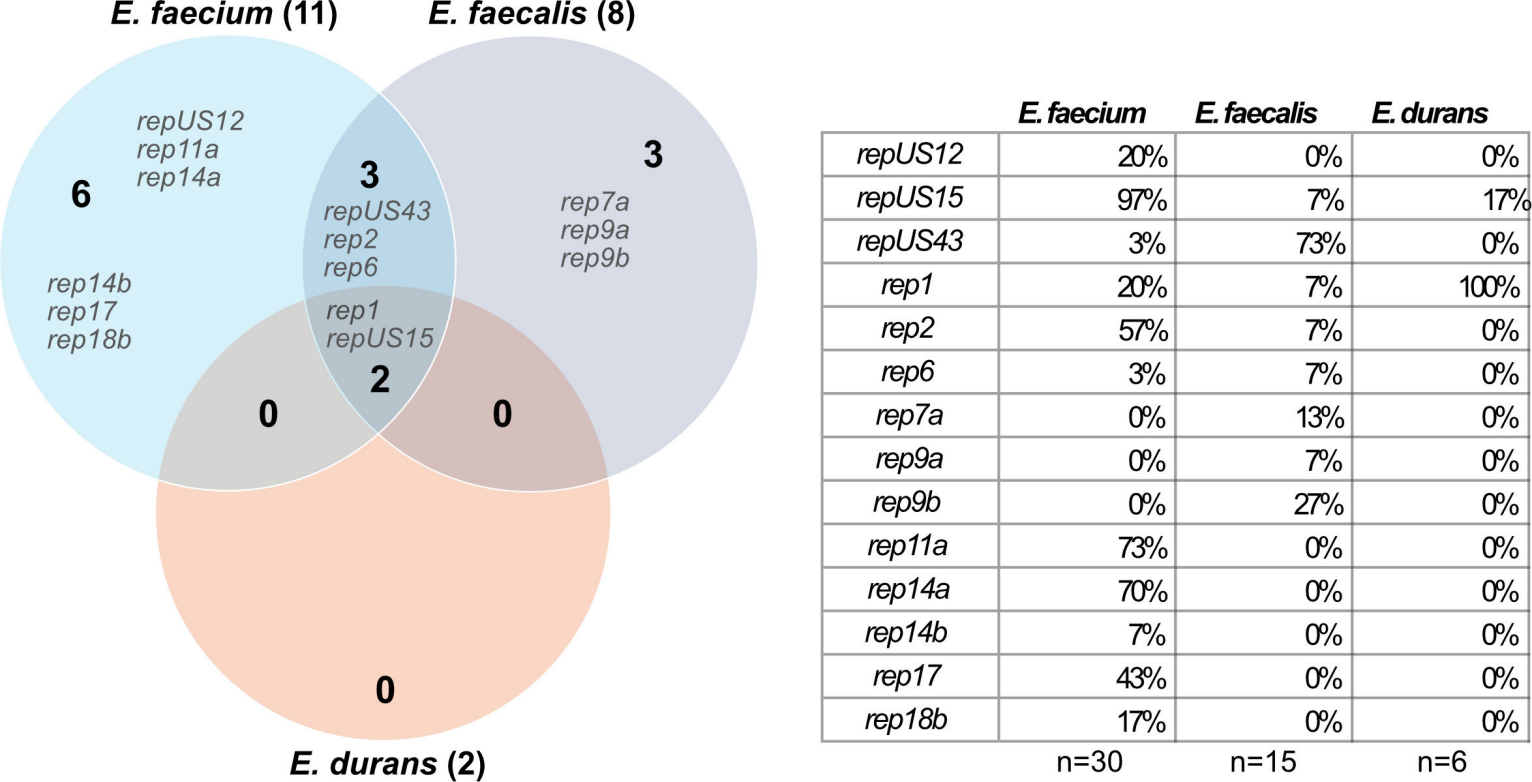
Enterococcal plasmids. Venn diagram illustrating species and indicated plasmids based on PlasmidFinder Rep-screen. The larger bold numbers within the circles represent the total number of unique plasmids carried by the species. The table details the percentages of sequenced isolates found with each given plasmid.

**Fig 3:**
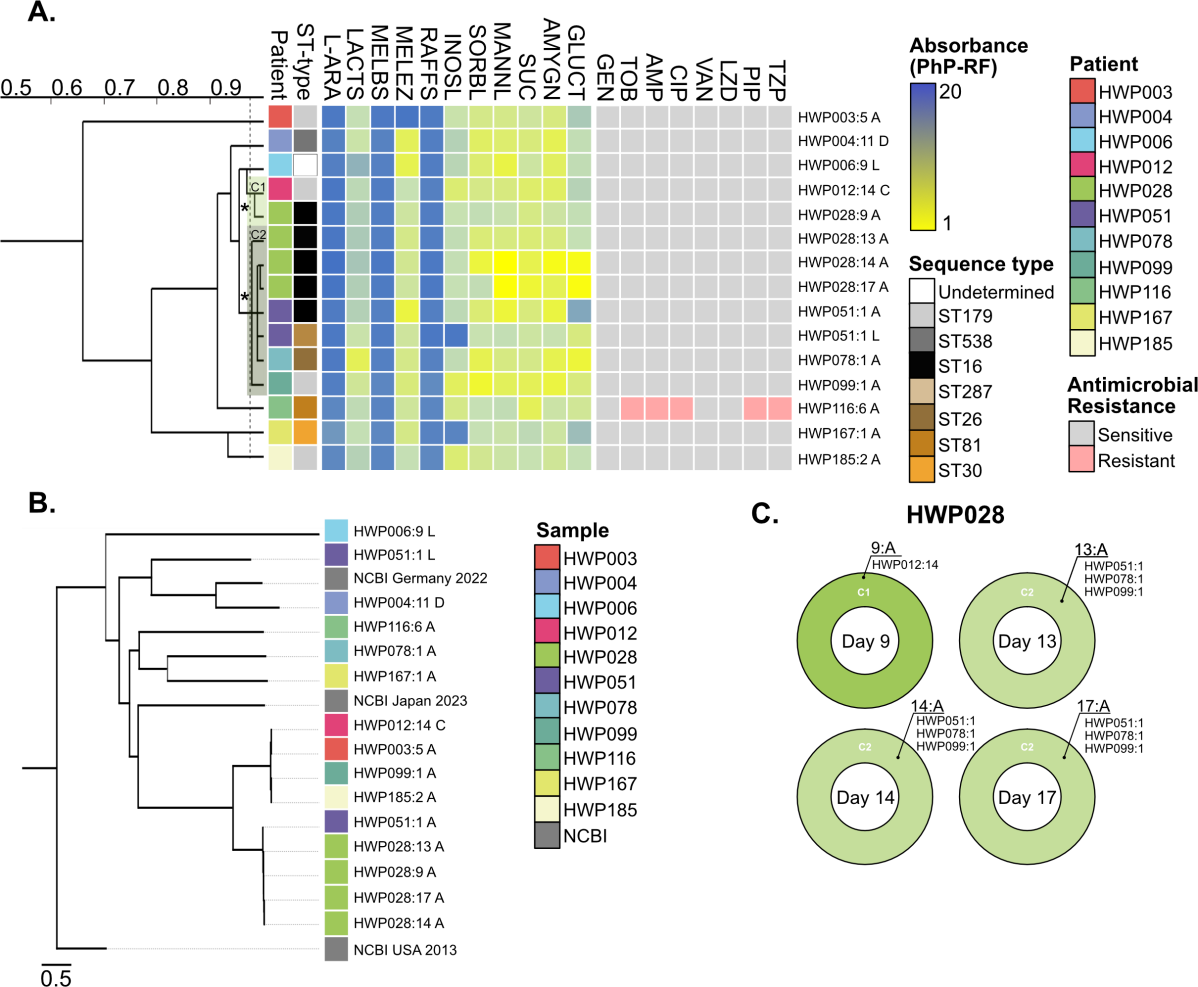
Heterogeneity of *E. faecalis* in the patient cohort. (A) Dendrogram derived from UPGMA clustering of PhP-RF data (absorbance values at 620 nm). The blue-yellow gradient represents a scale from low (blue) to high (yellow) metabolism. Isolates with the most recent branching point above the 0.975 cut-off value (dotted line) are grouped into a PhP-type. If more than one isolate per patient was isolated, these clusters were numbered within that species. If isolates from different patients were grouped into the same PhP cluster, this was indicated by an asterisk (*). Refer to the material and methods or supplementary data for a full list of metabolic reagents. Phenotypic resistance (pink) and susceptibility (gray) were assessed using broth microdilutions according to EUCAST. ST types were determined based on Illumina WGS. **(B)** Phylogenetic tree based on SNP comparison of Illumina WGS data. Three NCBI strains are included as comparative references based on diversified historical and geographic backgrounds. The tree was made using the NCBI US isolate (GCF_000393015) as the main reference. **(C) **PhP-type cluster diversity (proportion) is based on the number of colonies from the total number screened (*n* = 16) for isolates from patient HWP028.

*E. faecalis* formed several minor PhP-type clusters rather than major branches, and with only eight total PhP-types, the isolates were metabolically more similar to each other than what was observed among *E. faecium* isolates. Unlike *E. faecium*, none of the *E. faecalis* isolates were able to metabolize L-arabinose, melibiose, or raffinose (Fig. 3A). Many strains were proficient in metabolizing lactose, melezitose, inositol, sorbitol, mannitol, sucrose, amygdalin, and gluconate. HWP003:5A stood out as the most distinct isolate due to its inability to degrade melezitose. HWP167:1A demonstrated an inability to metabolize inositol (alone in this together with HWP051:L), accompanied by a reduced degradation of gluconate (shared only with HWP051:A). Isolate HWP006:9L exhibited reduced lactose metabolism and was the only isolate for which an ST type could not be determined. Additionally, HWP006:9L was the only *E. faecalis* strain carrying plasmids replicon repUS15 and rep1, otherwise only found among our *E. faecium* isolates (Fig. S3). Notably, patient HWP006 was colonized by *E. faecium* HWP006:5A four collection days prior, but those did not carry plasmid rep1; instead, they carried plasmid repUS12 (Fig. 2; Fig. S3). Multiple perfect MLST matches for *gdh* prevented definitive ST classification for HWP006:9L and its results should be interpreted with caution. HWP116:6A was the only *E. faecalis* isolate showing phenotypic resistance against important antibiotics (Fig. 3A). HWP116:6A only carried one plasmid replicon (repUS43) which was shared by most *E. faecalis* strains (Fig. S3). Only patient HWP028 was colonized by *E. faecalis* longitudinally (Fig. S1). HWP028:9-17 changed their metabolic fingerprint over time, gradually increasing the metabolic activity of sorbitol, mannitol, sucrose, amygdalin, and gluconate. This difference was significant enough to classify HWP028:9A as a separate PhP-type. There were no differences in plasmid content (Fig. S3), resistance genes (Fig. S5), or genotype (Fig. 3B). The second cluster (C2, Fig. 3C) in HWP028, represented by all strains from days 13 to 17, shared the cluster with isolates from three other patients, including isolates HWP051:1A/L, HWP078:1A, and HWP099:1A (Fig. 3A and C).

HWP051:1A and HWP051:1L had different ST types and grouped differently phylogenetically. Upon closer inspection, HWP051:1A (subpopulation 94% of total) carried significantly more AMR genes (Fig. S5), and a plasmid replicon (repUS43) that HWP051:1L did not. In fact, HWP051:1L was found with no plasmid at all, a trait shared only with HWP167:1A (Fig. S3). While it seems like the missing plasmid might explain the absence of AMR genes, repUS43 had not been associated with these AMR genes in any other of our isolates (Fig. 2). This information, coupled with the variation in ST type, indicates a heterogeneous population in patient HWP051.

### High heterogeneity was observed among *Enterococcus durans* isolates

**Fig 4:**
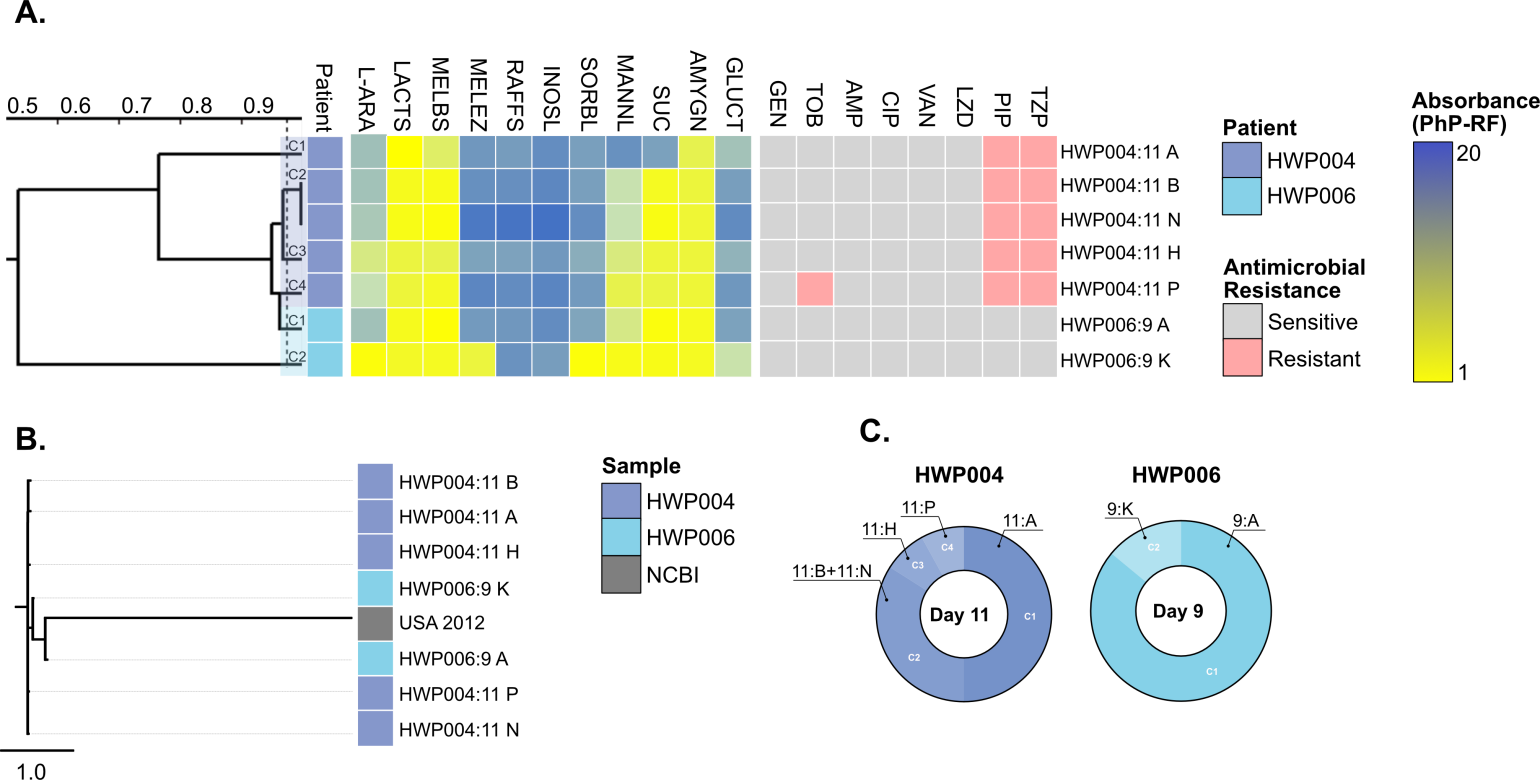
Heterogeneity of *E. durans*. Depiction of *E. durans* diversity in the patient cohort. (A) Dendrogram derived from UPGMA clustering of PhP-RF data (absorbance values at 620 nm). The blue-yellow gradient represents a scale from low (blue) to high (yellow) metabolism. Isolates with the most recent branching point above the 0.975 cut-off value (dotted line) are grouped into a PhP-type. If more than one isolate per patient was isolated, these clusters were numbered within that species (indicated by color). Refer to the material and methods or supplementary data for a full list of metabolic reagents. Phenotypic resistance (pink) and susceptibility (gray) were assessed using broth microdilutions according to EUCAST. (B) Phylogenetic tree based on SNP comparison of Illumina WGS data. One NCBI strain is included as a comparative reference. The tree was made using the NCBI US isolate (GCF_000407265) as the main reference. (C) PhP-type cluster diversity (proportion) is based on the number of colonies from the total number screened (*n* = 16) for isolates from patient HWP004 and HWP006.

For *E. durans*, the sample size was small (two patients), which is also reflected globally with very few strains deposited in NCBI, and it was in both instances co-isolated with *E. faecalis*. Overall, *E. durans* shared many metabolic traits with *E. faecium*, including poor metabolic activity for melezitose and inositol (Fig. 4A). None of the strains could metabolize raffinose, similar to *E. faecalis* and some *E. faecium* strains (Fig. S6). Sorbitol was well metabolized by *E. faecalis* (Fig. 3A) and over half of the *E. faecium* strains (Fig. 1A), but by none of the *E. durans* isolates. Importantly, all *E. durans* HWP004:11 strains were isolated on collection day 11, the same day both *E. faecium* HWP004:11E/L and *E. faecalis* HWP004:11D were isolated (Fig. S1). The *E. durans* isolates almost identically exhibited the phenotype of *E. faecium* HWP004:11E/L, except for *E. durans* HWP004:11A, which could not metabolize mannitol or sucrose (Fig. S6). In terms of AMR, all *E. durans* from patient HWP004 included resistance against piperacillin (PIP)/TZP, similar to *E. faecium*, with HWP004:11P additionally demonstrating resistance toward TOB, similar to *E. faecium* HWP004:11E. *E. durans* HWP006:9A/K were different from each other, with HWP006:9K capable of metabolizing L-arabinose (an *E. faecium* trait) and melezitose (an *E. faecalis* trait).

Indeed, this ability might be the reason why HWP006:9K grouped among *E. faecium* isolates instead of the remaining *E. durans* isolates (Fig. S6). None of the *E. durans* HWP006:9A/K demonstrated any phenotypic antibiotic resistance. Interestingly, *E. durans* HWP006:9A/K carried more resistance genes than any of the *E. durans* HWP004 isolates (Fig. S5). Notably, patient HWP006 had *E. faecium* strains isolated 4–5 collection days prior to *E. durans* HWP006:9A/K, and *E. faecalis* HWP006:9L isolated on the same day (Fig. S1). When instead looking at plasmids, the *E. faecium*, *E. faecalis,* and *E. durans* isolates from patient HWP006 all carried the repUS15 plasmid, marking the only potential occurrences of the repUS15 in *E. faecalis* and *E. durans*. Phylogeny indicated that all our *E. durans* strains were similar, but HWP006:9A/K stood out as the most different isolates (Fig. 4B). MLST could not be performed due to insufficient online resources for *E. durans*. Overall, the *E. durans* were metabolically diverse, and none of the strains survived within the patients for more than one collection day (Fig. S1).

## DISCUSSION

Differentiating species and clinically relevant strains of *Enterococcus*, often unobserved by conventional screening methods, is challenging and requires a fast and accurate screening scheme. In this study, we used the PhP-RF system, which provides a biochemical fingerprint for various clinical *Enterococcus* species, to investigate the heterogeneity of clinical *Enterococcus* during catheterized urine colonization.

Our study revealed considerable heterogeneity among *E. faecium* isolates. Although underexplored, particularly in the context of urinary infections, it has previously been demonstrated that *E. faecium* infections may involve multiple clones simultaneously (26, 27). Most isolates in our study belonged to the well-characterized clonal complex 17 (CC17, formerly known as clade A1), encompassing strains from ST127 (HWP004) and ST80 (HWP143, HWP199, HWP030, HWP072, HWP083, and HWP095). CC17 is widely recognized as the most significant cluster of *E. faecium*, often linked to enhanced biofilm formation, increased virulence, and AMR in healthcare settings (28, 29). Interestingly, HWP143:4:C was identified as ST22, which is not typically associated with pathogenicity (30), but has been proposed as an ancestral lineage of the CC17 cluster (31). Additionally, HWP006 isolates were classified as ST192, a sequence type recently identified as an emerging clone primarily in hospital settings in Germany (32).

Most isolates were classified into distinct PhP types. Patient HWP143 exhibited clear subpopulations due to the presence of multiple ST types. In other strains, particularly those from longitudinal samples, metabolic variations may suggest metabolic adaptations to the bladder/catheter environment. *Enterococci* are generally thought to lack the necessary enzymes to metabolize urine-based carbon and nitrogen sources, such as creatine and urea (33, 34). However, patients in ICUs with conditions such as diabetes or acute kidney injury (like those in our cohort) may excrete unaltered carbohydrates, including glucose, lactose, and L-arabinose (35), and this could speculatively vary further in the microenvironments of a catheter. Mannitol is sometimes administered as a diuretic in clinical settings (36); however, this was not the case in our cohort. Isolates from both HWP004 and HWP030 exhibited increased metabolic activity of L-arabinose over time. For patient HWP143, the majority of the *E. faecium* population (81%) belonged to the non-pathogenic ST22, suggesting that the presence of CC17 most likely would have been overlooked by conventional screening methods (6).

In patient HWP030, a metabolic shift was observed between collection days 5 and 6, despite genetic similarity across the isolates, with further alterations leading to a loss of CIP resistance in 93% of the population by day 8. Across *E. faecium*, there were variations in AMR within patient isolates, specifically against TOB (HWP004 and HWP006), PIP/TZP (HWP004), and CIP (HWP030), as well as differences in AMR genes (HWP004 and HWP143). Although none of these antibiotics are first-line treatments for *E. faecium*, this finding adds to the growing body of evidence of AMR variability among heterogeneous populations within an infection (37). Indeed, even when the dominant populations were initially resistant, the resistant phenotype rapidly diminished in subsequent collection days, likely due to the absence of selective pressure, at least to a prevalence below our detection threshold.

Plasmid carriage varied among isolates from the same patient, independent of changes in AMR. On collection day 11, the dominant *E. faecium* population of HWP004 (represented by HWP004:11E) carried rep1 but lacked repUS15, while a minor subpopulation (represented by HWP004:11L) carried both plasmids. By day 12, rep1 was absent in two of our strains (HWP004:12A/F) but reappeared in all strains by day 13, potentially maintained by isolate HWP004:12B. Upon closer investigation of HWP004:12B, it was observed that the two replicon-plasmids were located on what seemed to be a hybrid plasmid (Fig. S4), but this was not investigated further. Previous studies have identified rep1 plasmids as conjugative plasmids specific to *E. faecalis*, associated with AMR and virulence, and typically absent in *E. faecium* (Table S2) (38, 39). Interestingly, all five HWP004:11 *E. durans* isolates carried this plasmid. This was not the only occurrence of the rep1 plasmid replicon, as it was additionally detected in HWP143:4C. Another notable finding was the presence of rep6 in HWP072:4A. Although this plasmid is small and cryptic, it has been proposed as an *E. faecalis*-specific plasmid (38). For isolates in patients HWP030, HWP083, and HWP199, significant nucleotide variations were observed in rep11a (suggested to be a toxin-related plasmid, Table S2) and rep14a (small and cryptic), as these plasmid replicons showed deviations in identity level between collection days, indicating potential environmental adaptation through plasmid rearrangement.

*E. faecalis* isolates exhibited limited heterogeneity when assessing ST types within a patient but demonstrated notable diversity in both ST type and metabolic profile across different patients. *E. faecalis* ST types are generally less homogeneous, likely due to their widespread ecological distribution, which contributes to the limited understanding of the significance of specific ST types (40). The predominant ST type identified was ST179 (HWP003, HWP012, HWP099, and HWP085), which, along with ST16 (HWP028 and one isolate from HWP051), belongs to clonal complex 58 (41). CC58 has been frequently associated with clinical infections, particularly in ICUs, and with AMR (41, 42). Other identified ST types included ST538 (HWP004 and CC241), ST287 (one isolate from HWP051), ST26 (HWP078), ST81 (HWP116), and ST30 (HWP167), all ST types of which have previously been linked to clinical infections (43–47). The stand-out example of heterogeneity among *E. faecalis* was observed in patient HWP051, who was colonized by both ST16 (94% subpopulation, represented by HWP051:1A) and ST287 (6% subpopulation, represented by HWP051:1L) simultaneously. While prior research on *E. faecalis* heterogeneity has often focused on strain properties, different patient samples, or outbreak scenarios (17, 48, 49), the occurrence of mixed enterococcal site infections has not been extensively documented. The rarity of reporting mixed infections might be due to the challenges in distinguishing species, as well as the fact that screenings rarely encompass as many colonies as our current study for the investigation. Indeed, in our previous screen where 10 colonies were selected, and the batch was later assessed by MALDI-TOF, we did not detect *E. faecalis* HWP004, even though *E. faecalis* had been isolated from the blood of the same patient during our urine collection period (6) (patient A in cited paper). This underscores that more pathogenic strains might be missed due to the lack of thorough investigations.

In contrast to *E. faecium*, *E. faecalis* isolates did not metabolize L-arabinose, melibiose, or raffinose, but efficiently utilized other carbon sources such as lactose, melezitose, inositol, sorbitol, mannitol, sucrose, amygdalin, and gluconate. These findings align with established knowledge of *E. faecalis* metabolism (50), reinforcing the species-specific metabolic pathways that have been previously documented. Notably, HWP003:5A lacked the ability to degrade melezitose, while HWP167:1A showed deficiencies in metabolizing inositol and gluconate. The isolates from patient HWP051 exhibited distinct metabolic profiles, with key differences in their ability to degrade melezitose, inositol, mannitol, amygdalin, and gluconate. Strains from patient HWP028 demonstrated increased metabolic activity for mannitol, sucrose, amygdalin, and gluconate over time, suggesting the potential importance of these carbon sources during *E. faecalis* urine or catheter colonization (6, 51, 52).

Most *E. faecalis* isolates harbored the repUS43 plasmid, a conjugative plasmid previously identified in only one of our *E. faecium* isolates. The isolate *E. faecalis* HWP006:9L seemed to carry the repUS15 plasmid, suggesting possible HGT from *E. faecium*. Interestingly, this plasmid was also found in one *E. durans* isolate from the same isolation day. The repUS15 plasmid has recently been described as a carrier for high-level aminoglycoside resistance and as a potential carrier for *vanA*-mediated VAN resistance (53, 54). HWP078:1A possessed the highest number of plasmid replicons (six), including one (rep7a) not typically associated with enterococcal plasmids (Table S2) (38). Additionally, *E. faecalis* HWP116:6A was the only isolate exhibiting phenotypic resistance to TOB, AMP, CIP, PIP, and TZP, despite carrying few AMR genes. HWP116:6A only carried the repUS43 plasmid replicon, which on further investigation turned out to be chromosomally integrated (Fig. S4). This case of multidrug-resistant *E. faecalis* is particularly concerning given the general characterization of *E. faecalis* as more virulent but less resistant than *E. faecium* (55). Unlike *E. faecium*, *E. faecalis* plasmids did not exhibit nucleotide changes over time. We could not identify any longitudinal changes in AMR or AMR genes; the location of these genes remains unexplored in the current study. Two of the *E. faecalis* strains were not found to carry any plasmid at all.

*E. durans* isolates, though less common, shared several metabolic features with *E. faecium*, such as poor activity for melezitose and inositol metabolism and an inability to metabolize raffinose. Unlike *E. faecalis* and many *E. faecium* strains, none of the *E. durans* isolates could metabolize sorbitol. Two *E. faecium* strains were initially misidentified as *E. durans* by MALDI-TOF but were confirmed as *E. faecium* through sequencing. This misidentification could only partly be resolved by PhP-RF and is consistent with previous reports highlighting the similarity between *E. durans* and *E. faecium* (56). Despite being prevalent for only one day, these findings are significant as *E. durans* has been associated with higher rates of progression from urinary tract infections to bacteremia (57, 58). Interestingly, speculated events of HGT might have occurred in patients co-colonized with *E. durans* (HWP004 and HWP006). However, these patients also harbored *E. faecium* and *E. faecalis* with undetermined ST types, making it impossible to distinguish contributing species. While further molecular and genomic investigations are required to prove the phenomenon, our *E. durans* HWP006:9A might be a case of interspecies plasmid conjugation.

The *E. durans* strains from patient HWP004, isolated on the same day as *E. faecium* HWP004:11E/L and *E. faecalis* HWP004:11D, resembled the *E. faecium* HWP004:11E/L phenotype, except for *E. durans* HWP004:11A, which could not metabolize mannitol or sucrose. Isolates of *E. durans* from patient HWP004 also exhibited a similar AMR phenotype to *E. faecium*. In contrast, *E. durans* HWP006 isolates displayed unique metabolic capabilities and carried more resistance genes than isolates from patient HWP004. However, no phenotypic AMR was observed, similar to co-isolated *E. faecalis* isolates. Phylogenetically, *E. durans* strains from patient HWP004 were metabolically similar, while the HWP006 isolates stood out both from each other and from the other *E. durans* isolates, underscoring significant heterogeneity among *E. durans*. Notably, all strains carried the rep1 plasmid, which has previously been suggested to be associated with *E. faecalis* (38), indicating a potential similarity between these two species.

We acknowledge several limitations in this study. Colony-forming unit (CFU) counts from frozen urine are not as optimal as using freshly collected urine. Despite our initial control experiments, the freezing procedure may have impacted different species of *Enterococcus* to varying degrees. This challenge is encountered by researchers working with biobanks and in microbiome research. *Enterococcus* spp. from complex clinical catheter samples are often polymicrobial. Although colonies were restreaked for purity, species were confirmed by MALDI-TOF, and basic local alignment search tool (BLAST) was performed on the same confirmed colony, we acknowledge that low-level contamination cannot be entirely excluded. The close phenotypic similarity in *Enterococcus*, combined with horizontal gene transfer, makes it inherently difficult to distinguish low-level contamination from genuine interspecies exchange. MLST classification was also inconclusive for two isolates due to incomplete or ambiguous allele calls. We therefore interpret such findings as hypothesis-generating rather than conclusive. We hope that the techniques employed in this study will help guide future research in developing effective methods for assessing bacteriuria.

### Conclusion

This research provides significant insights into the heterogeneity and metabolic diversity of *Enterococcus* species during CAUTIs, particularly *E. faecium* and *E. durans*, across various dimensions. Our findings demonstrate considerable genetic and metabolic diversity among *E. faecium* isolates, with most belonging to CC17, a group known for its enhanced virulence and antibiotic resistance. Unlike earlier studies that focused primarily on single lineages, we identified the presence of multiple lineages and ST types within individual patients, including ST22, an ancestral lineage of CC17, and ST192, an emerging clone in hospital settings. Notably, metabolic adaptations were observed, such as increased metabolism of L-arabinose, and significant shifts in antibiotic resistance patterns over time. Our work reveals a previously underappreciated metabolic diversity.

*E. faecium* isolates exhibited variability in plasmid content and types. For instance, the rep1 plasmid, typically associated with *E. faecalis*, was found in both *E. faecium and E. durans*, hinting at HGT. Variations in plasmid nucleotide sequences over time were also observed, which could reflect environmental adaptation. *E. faecalis* isolates showed less genetic heterogeneity but displayed notable diversity in metabolic profiles across different patients. Evidence of mixed colonization with different ST types and variations in antibiotic resistance was found. Plasmids such as repUS43 and repUS15, plasmids not typically associated with *E. faecalis*, were identified, further highlighting the complexity of these infections. *E. durans* isolates, though less common, exhibited metabolic similarities to *E. faecium*. The misidentification of *E. durans* as *E. faecium* by MALDI-TOF, later confirmed through sequencing, underscores the need for accurate identification methods.

This research reveals that PhP-RF can be used to distinguish primarily clinical *E. faecium* and *E. faecalis* from each other, and that *E. durans* might metabolically group among *E. faecium* isolates. We have given insight into the dynamic nature of *Enterococcus* from urinary catheters, emphasizing significant variability in genetic and metabolic profiles both within individual patients and over time. The study offers a novel understanding of plasmid diversity among these species, which may have important implications for our understanding of antibiotic resistance and virulence. These findings underscore the critical need to consider metabolic adaptations and genetic heterogeneity, particularly in clinical settings where conventional screening methods may overlook the presence of multiple clones and lineages when studying *Enterococcus* infections, as they will come to impact disease severity and treatment outcome.

## MATERIALS AND METHODS

### Sample collection

Urine sample collection was done as previously described (6, 25). Briefly, urine was collected from the catheter into sterile vacutainer tubes and transported cold. All urine samples were stored in cryovials with 10% dimethyl sulfoxide at −80°C. The same study performed a full screen of all urine samples and collection days from 210 patients. If a sample turned out positive for *Enterococcus*, it was screened again to assess heterogeneity. To ensure that CFU assessment of *Enterococcus* was unaffected by freezing, a small subset of patients (*n* = 12) was controlled for bacterial colonization both before and after freezing. This current study focused on *Enterococcus*-positive urine samples (*n* = 29) from the initial screen, from which 16 colonies per urine sample (*n* = 464) were further investigated. These 29 urine samples were collected from 17 patients (Fig. S1).

### *Enterococcus* identification and isolation

For *Enterococcus* identification, 100 µl of thawed urine was streaked on Brilliance UTI Clarit agar (UTI agar) plates, incubated for 24 h at 37°C. Urine was diluted in phosphate-buffered saline to meet *Enterococcus* CFU around 50–100 CFU per plate. To investigate heterogeneity, 16 *Enterococcus* colonies were picked from each plate and re-streaked on brain heart infusion (BHI) agar, incubated at 37°C for 24 h. If the number of single *Enterococcus* colonies was less than 16, or the urine contained polymicrobial colonization complicating collection, supplementary streaking on UTI agar was performed using additional urine. This step was to ensure the isolates were pure *Enterococcus* colonies. The 16 colonies could constitute either one *Enterococcus* species or multiple. Cluster fractions and percentages were calculated on the number of colonies within a given species among the 16 colonies, hence 100% for one species could mean less than 16 colonies if they were split among several *Enterococcus* spp. on that day.

### PhP-RF system screening

The PhP-RF system is a rapid phenotypic screening method from the PhenePlate typing system assessing the kinetics of biochemical reactions involved in prokaryotic metabolism. It is composed of a 96-well microtiter plate with 11 different dehydrated substrates coated at the bottom (Table S1). In our study, all 16 isolates per urine sample were tested using the PhP-RF system. After three readings of absorbance, the mean value was calculated to compare similarities. Pairwise similarities between isolates showed their biochemical fingerprints and were calculated as correlation coefficients (23). These correlation coefficients were used by the PhPWIN software to create a similarity matrix, later clustered into a dendrogram using UPGMA. Isolates sharing biochemical fingerprint similarity below 0.975 were recognized as different PhP-types indicating metabolic heterogeneity.

A suspending medium containing 0.011% bromothymol blue, 0.2% proteose peptone, 0.05% yeast extract, 0.5% sodium chloride, and a 0.2M solution of phosphate buffer (Na_2_HPO_4_ + NaH_2_PO_4_) at pH 7.5 was prepared according to the instruction manual of the PhP-RF system (18). *Enterococcus* colonies from BHI agar were picked with sterile toothpicks and added to the first column of the PhP-RF plate and then aliquoted from column one to each of the reagent wells in the consecutive columns. The inoculated PhP-RF plates were incubated inside a moist microaerophilic chamber for 64 h at 37°C. Absorbance at 620 nm was measured after 16, 40, and 64 h with a Spark 10M Multimode Microplate Reader (Tecan Nordic AB). The tested compounds included L-arabinose (L-ARA), lactose (LACTS), melibiose (MELBS), melezitose (MELEZ), raffinose (RAFFS), inositol (INOSL), sorbitol (SORBL), mannitol (MANNL), sucrose (SUC), amygdalin (AMYGN), and gluconate (GLUCT).

### Antimicrobial resistance testing

The AMR tests were carried out for a selection of clinically relevant antibiotics in round-bottom 96-well plates. Concentrations were only tested at the clinical breakpoint provided by EUCAST (v 14.0)(59). Bacterial suspensions were calibrated in 0.9% NaCl to 0.5 McFarland standard units, diluted in Mueller Hinton (MH) broth, and added to each well at a final concentration of 5 × 10^5^ cells/ml. Positive control wells had no antibiotics (growth control), and negative control wells had no bacteria (media control). Both positive and negative control wells were included in every plate. Antibiotics used included gentamicin, TOB, AMP, CIP, VAN, linezolid, PIP, and TZP. Tazobactam concentration was added as 4 mg/L for all TZP wells. The plates were incubated for 18–20 h at 37°C.

### Strain identification and WGS

A minimum of one representative isolate per PhP-type was used for strain identification. Fresh colonies on BHI plates were identified with MALDI-TOF (Bruker Biotyper), additionally confirmed by WGS. The same colonies used for MALDI-TOF were suspended in BHI broth and prepared as overnight cultures in arrangement for DNA extraction (*E. faecium*: *n* = 31, *E. faecalis*: *n* = 18, and *E. durans*: *n* = 5). DNA extraction was performed on overnight cultures using Lucigen MasterPure Complete DNA and RNA Purification Kit (Cat. No. MC85200). DNA quantity was controlled with a Qubit 2.0 Fluorometer for broad rage double-stranded DNA. Extracted DNA was sent to BMKGENE for Illumina-based (DNBseq, pair-ended short-insert read, 150 bp read length) WGS. For a subset of strains, we carried out complementary Oxford Nanopore long-read sequencing (rapid barcoding kit 24, V.14, SQK-RBK114.24, PromethION R10.4.1 flow cell), later corrected using the Illumina reads.

### Sequence analysis

Sequence analysis was performed in CLC Genomics Workbench v24.0.1, as described in our lab (60). Selected isolates belonging to different PhP types were sequenced by Illumina, and reads were paired, trimmed, and assembled into contigs. Species were confirmed using the NCBI nucleotide BLAST on the largest contigs assembled. To compare isolates genetically, we crafted a phylogenetic tree using SNP-inferred phylogeny from CSI Phylogeny 1.4, standard settings (61). Phylogenetic trees were generated per species, and while several NCBI strains were included for comparative purposes, the main references used to create the trees were GCF_002007625 (*E. faecium*), GCF_000393015 (*E. faecalis*), and GCF_000407265 (*E. durans*). ST of our strains were determined using multi-locus sequence typing on *de novo* assembled genomes (MLST 2.0) (21, 62–67). ResFinder v4.1 was used to identify AMR genes based on unassembled reads (68), and Plasmid Finder (v2.1) was used as an indication for the presence of plasmids (69). All resistance genes identified by ResFinder were presented. A subset of strains with plasmids was Nanopore sequenced and assembled (Long Read Support v24.0), and confirmed plasmids were aligned (Whole Genome Alignment V24.0) to reference plasmids (NCBI, BLAST) in CLC Genomics Workbench.

### Visualization

PhP-type trees were generated with the PhenePlate software while phylogenetic SNP trees were generated with CSI Phylogeny 1.4. AMR profile illustrations were created using Microsoft Excel (v. 16.86). Illustrations for PhP types within a patient (circle diagrams) were created using GraphPad Prism (v. 10.2.3). Venn diagram and combination of data sets into single figures were performed using Affinity Designer (Serif/Affinity, v. 1.10.0). Hybrid plasmid was constructed and visualized in CLC Genomics Workbench.

## Data Availability

The study cohort data set can be found under the SciLifeLab Data Repository (14229410) while available sequences have been deposited at NCBI (PRJNA1187867).

## SUPPLEMENTAL MATERIAL

Supplemental tables and figures spectrum.03160-24-s0001.docx Tables S1 to S4 and Fig. S1 to S6.

ASM does not own the copyrights to Supplemental Material that may be linked to, or accessed through, an article. The authors have granted ASM a non-exclusive, world-wide license to publish the Supplemental Material files. Please contact the corresponding author directly for reuse.
