# Supplementary material for: Heterogeneity and metabolic diversity among Enterococcus species during long-term colonization
Source: Microbiol Spectr. 2025 Jun 12;13(8):e03160-24. doi: 10.1128/spectrum.03160-24 (PMC12323598; doi:10.1128/spectrum.03160-24)
Supplement: Supplemental tables and figures — Tables S1 to S4 and Fig. S1 to S6. [file spectrum.03160-24-s0001.docx]

# **SUPPLEMENTARY**

**Supplementary Table 1. Carbon sources**

| L-arabinose | L-ARA | Monosaccharide found in e.g. dairy |
| --- | --- | --- |
| Lactose | LACT | Disaccharide found in e.g. dairy |
| Melibiose | MELBS | Disaccharide found in e.g. legumes, byproduct of raffinose |
| Melezitose | MELEZ | Trisaccharide found in e.g. tree sap, tree fungus or honeydew |
| Raffinose | RAFFS | Trisaccharide found in e.g. plant tissue |
| Inositol | INOSL | Sugar alcohol found in e.g. whole grain, fruits and nuts |
| Sorbitol | SORBL | Sugar alcohol found in e.g. fruits, sugar-free products and processed food |
| Mannitol | MANNL | Sugar alcohol found in e.g. mushrooms, vegetables, sugar-free products and processed food |
| Sucrose | SUC | Disaccharide found in e.g. sugarcane and fruits |
| Amygdalin | AMYGN | Cyanogenic glycoside found in the e.g. seeds of certain fruits |
| Gluconate | GLUCT | Conjugate base of organic base found in berries and fermented food |


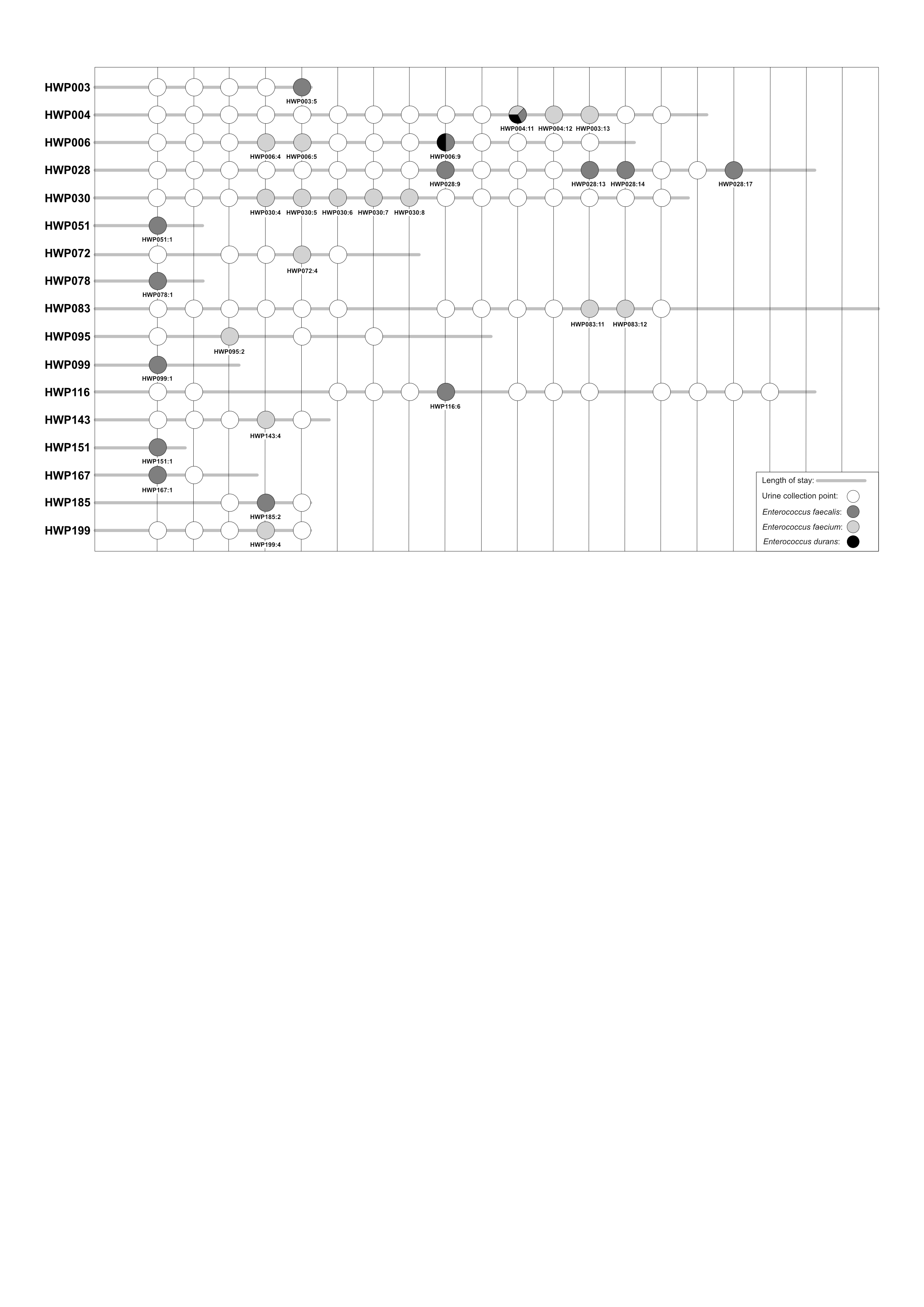


**Supplementary Figure 1.** Timeline for included patients. Length of stay is indicated by a gray bar along the X-axis, and sampling is marked by circles (possible collection points marked by vertical lines). Collections where *Enterococcus* spp. were present are marked in grayscale (*E. faecalis*: dark grey, *E. faecium*: light grey, *E. durans*: black). No growth is marked with empty circles.


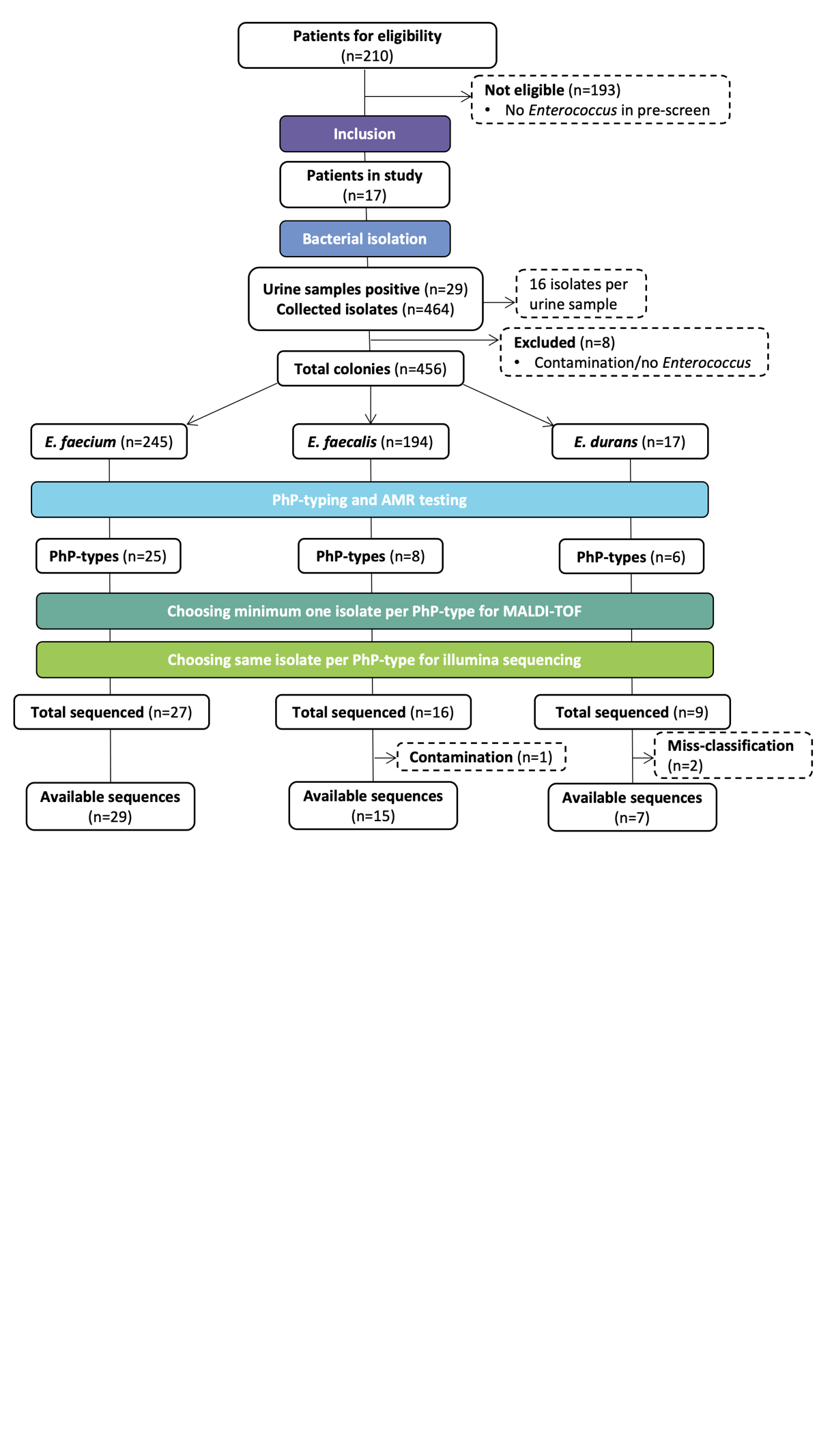


**Supplementary Figure 2.** Illustrative pipeline of sample processing from patient eligibility to final count of included sequences.


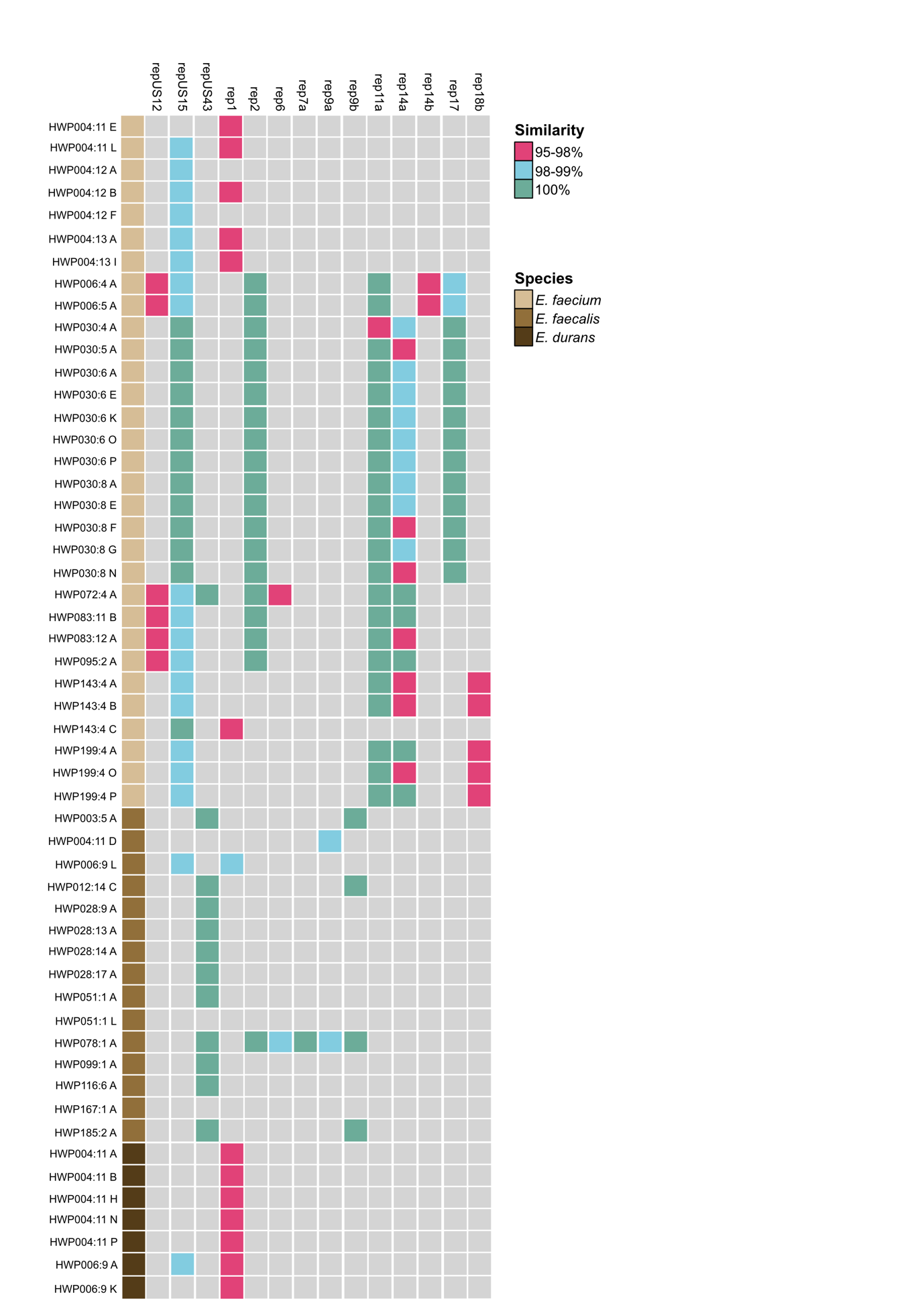


**Supplementary Figure 3.** Illustration of plasmid content as identified by PlasmidFinder. Color indicates presence (red, blue, green) and no presence (gray) of plasmid. Species and strains (left) and plasmid replicons (top) are indicated.

Among the plasmids we identified, two were particularly interesting. The first was from *E. faecium* HWP004:12B, where two plasmid replicons, rep1 and repUS15, were initially identified. Following long read assembly, only four contigs were identified: three belonging to the chromosome (total size 2,527,854 bp) and one representing a hybrid plasmid formed from rep1 and repUS15 (112,890 bp). We subsequently conducted whole genome alignment of this hybrid plasmid contig with two plasmids having the highest similarity according to BLAST.


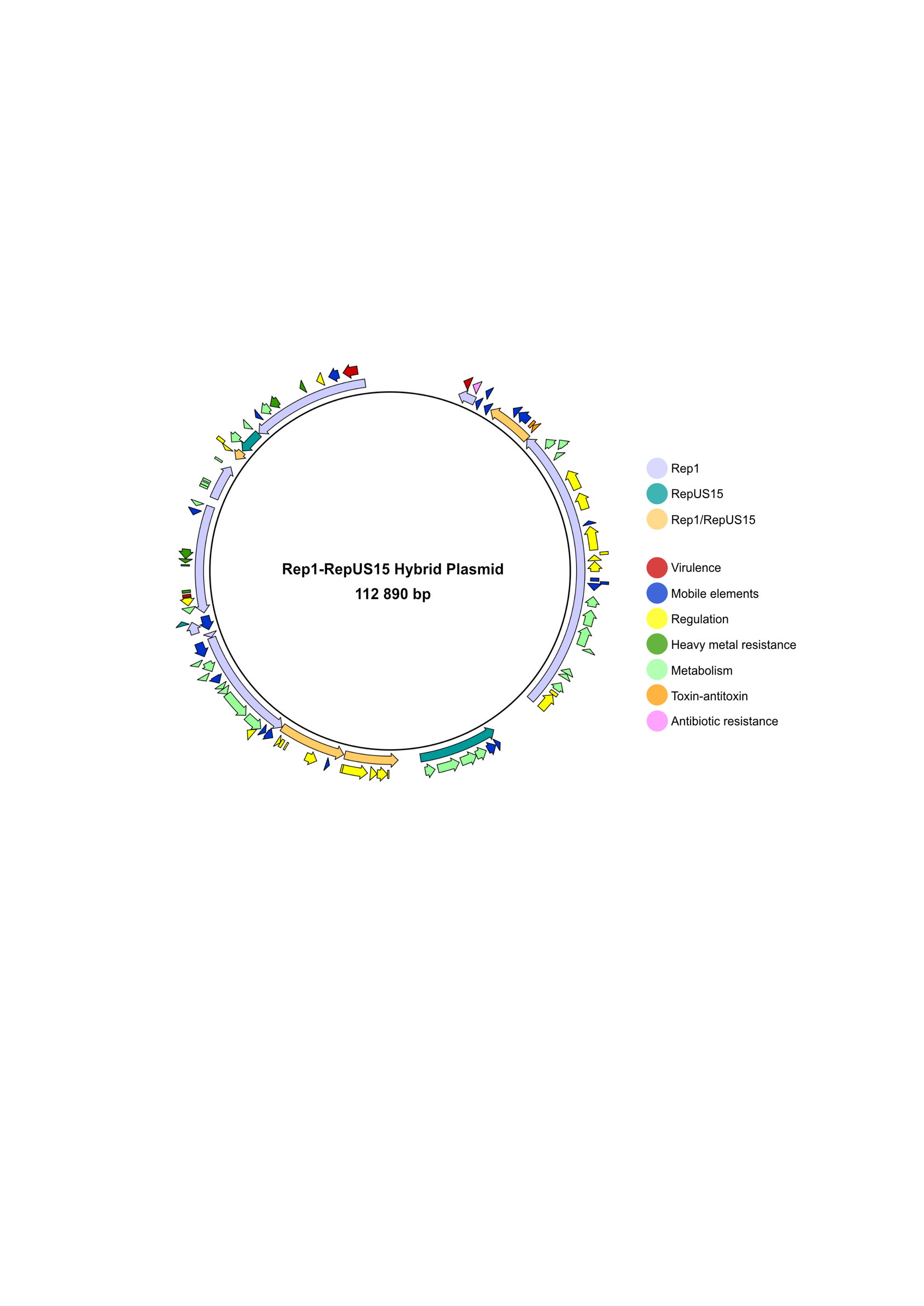
The second notable strain was from HWP116:6A, where initially, the repUS43 replicon was identified. After assembly of long read data, only one single contig could be identified (3,001,122 bp). A whole-genome alignment of the plasmid and the bacterial chromosome illustrate that the plasmid appears to have integrated into the chromosome (data not shown).

**Supplementary Figure 4.** Hybrid plasmid from *E. faecium* HWP004:12B annotated in CLC Genomics Workbench using plasmid sequences from both rep1 and repUS15 plasmids (CP016164 and CP004064). Certain parts of the plasmids are shared and indicated in our plasmid by bright orange (Rep1/RepUS15). Colors indicate suggested functional gene properties based on information received from annotated plasmids.

**Supplementary Table 2. Enterococcal plasmids**

| **Replicon** | **Alternative name** | **Hosts** | **Characteristic** | **Host range** |
| --- | --- | --- | --- | --- |
| repUS12 | - | - | - | - |
| repUS15 | RepA_N | - | Conjugative T4SS | Narrow |
| repUS43 | Rep_trans | - | Conjugative T4SS | - |
| rep1 | Inc18 | *E. faecalis*  Other G+ | Toxins  AMR  Conjugative | Broad |
| rep2 | Inc18 | Enterococci | Toxins  AMR  Conjugative | Narrow |
| rep6 |  | *E. faecalis*  Other G+ | Small, cryptic  Rolling circle | Broad |
| rep7a | pT181 Class 1 | Other G+ | AMR  Small | Broad |
| rep9a | RepA_N | *E. faecalis* | Sex-pheromone  AMR  Virulence | Narrow |
| rep9b | RepA_N | *E. faecalis* | Sex-pheromone  AMR  Virulence | Narrow |
| rep11a | Rep3 | Enterococci | Toxins | Narrow |
| rep14a | Rep_trans | *E. faecium* | Conjugative  Small, cryptic | Narrow |
| rep14b | Rep_trans | *E. faecium* | Conjugative  Small, cryptic | Narrow |
| rep17 | RepA_N | *E. faecium* | Conjugative  AMR | Narrow |
| rep18b | Rep3 | Enterococci | Unknown | Narrow |

Data in this table is based on previous studies (38, 39, 70, 71).


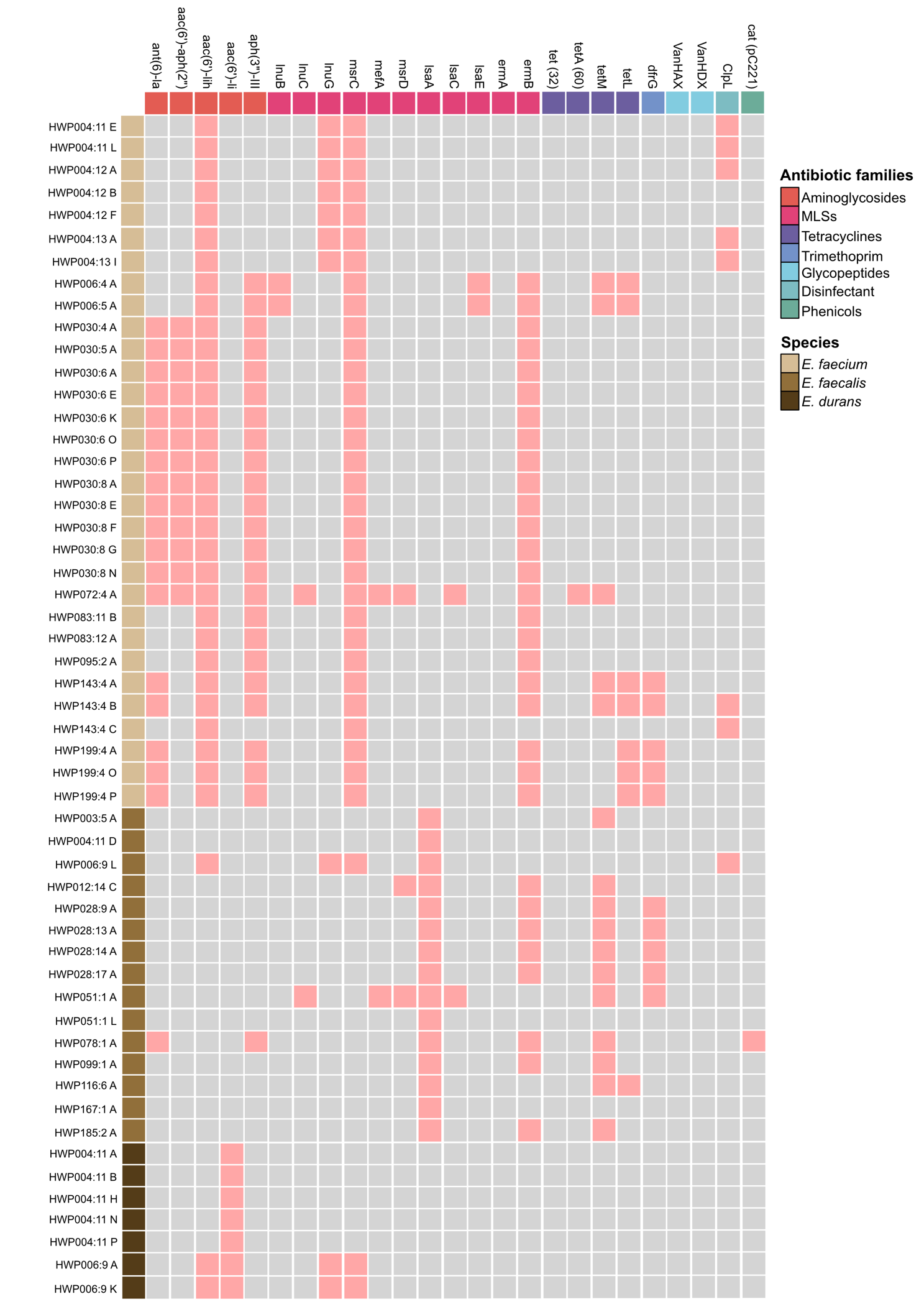


**Supplementary Figure 5.** Illustration of all antibiotic resistance genes as identified by ResFinder. Color indicates presence (pink) and no presence (gray) of the corresponding gene. Species and strains (left) and resistance gene with the corresponding antibiotic family grouping (top) are indicated.


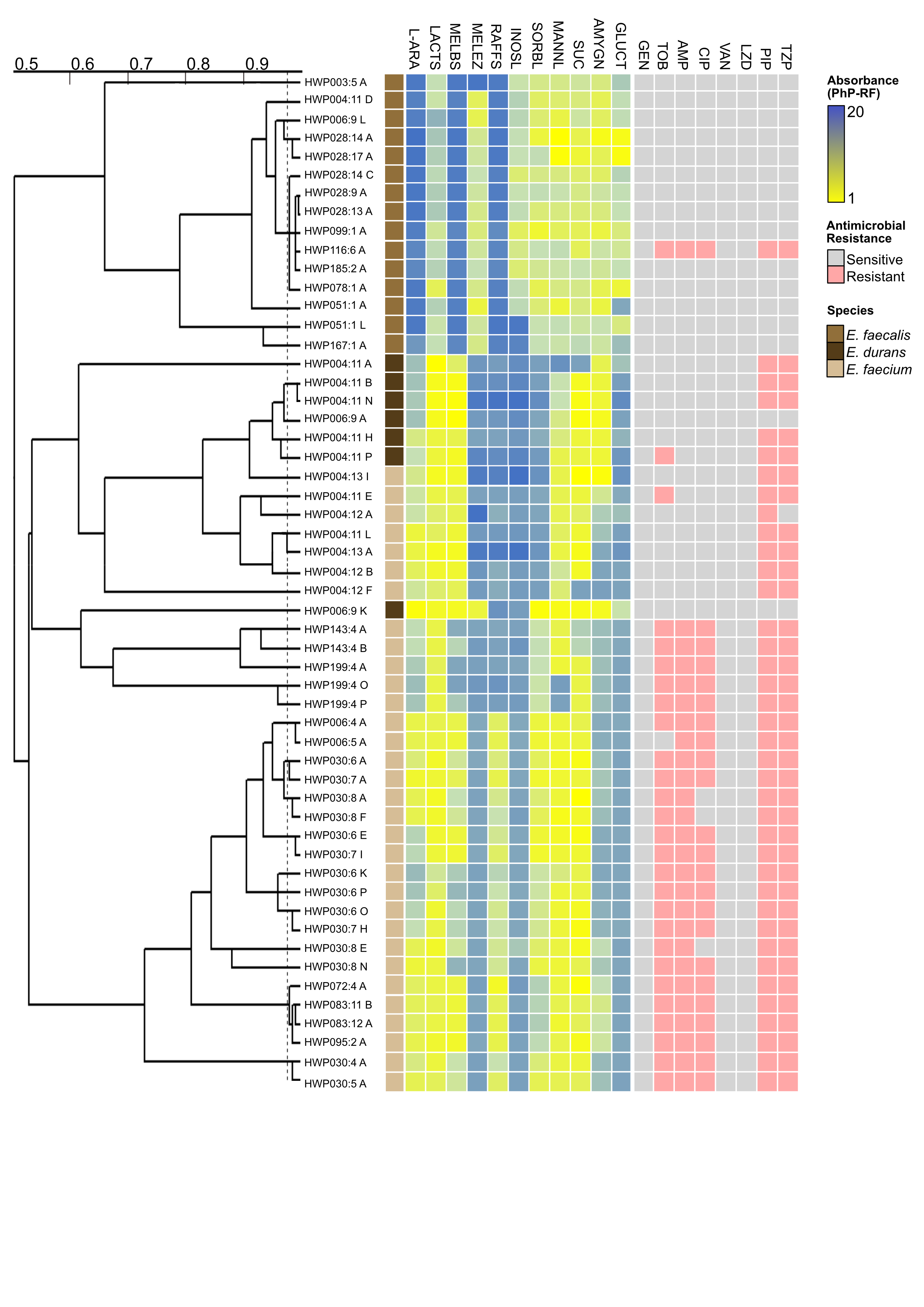


**Supplementary Figure 6.** PhP types as previously presented. All species combined into one PhP tree for interspecies comparison.
